# Thyroid hormone levels associate with exposure to polychlorinated biphenyls and polybrominated biphenyls in adults exposed as children

**DOI:** 10.1186/s12940-019-0509-z

**Published:** 2019-08-23

**Authors:** Sarah W. Curtis, Metrecia L. Terrell, Melanie H. Jacobson, Dawayland O. Cobb, Victoria S. Jiang, Michael F. Neblett, Sabrina A. Gerkowicz, Jessica B. Spencer, M. Elizabeth Marder, Dana Boyd Barr, Karen N. Conneely, Alicia K. Smith, Michele Marcus

**Affiliations:** 1 Emory University School of Medicine, 101 Woodruff Circle NE, Ste 2205A, Atlanta, GA 30322 USA; 2 Emory University Rollins School of Public Health, 1518 Clifton Rd, Atlanta, GA 30322 USA; 3 Emory University School of Medicine, 615 Michael St, Atlanta, GA 30322 USA

**Keywords:** Endocrine disrupting compound, EDC, PBB, PCB, Age at exposure, Children’s health, Triiodothyronine, Thyroxine, Thyroid-stimulating hormone, DOHaD

## Abstract

**Background:**

Michigan residents were directly exposed to endocrine-disrupting compounds, polybrominated biphenyl (PBB) and polychlorinated biphenyl (PCB). A growing body of evidence suggests that exposure to certain endocrine-disrupting compounds may affect thyroid function, especially in people exposed as children, but there are conflicting observations. In this study, we extend previous work by examining age of exposure’s effect on the relationship between PBB exposure and thyroid function in a large group of individuals exposed to PBB.

**Methods:**

Linear regression models were used to test the association between serum measures of thyroid function (total thyroxine (T_4_), total triiodothyronine (T_3_), free T_4_, free T_3_, thyroid stimulating hormone (TSH), and free T_3_: free T_4_ ratio) and serum PBB and PCB levels in a cross-sectional analysis of 715 participants in the Michigan PBB Registry.

**Results:**

Higher PBB levels were associated with many thyroid hormones measures, including higher free T_3_ (*p* = 0.002), lower free T_4_ (*p* = 0.01), and higher free T_3_: free T_4_ ratio (*p* = 0.0001). Higher PCB levels were associated with higher free T_4_ (*p* = 0.0002), and higher free T_3_: free T_4_ ratio (p = 0.002). Importantly, the association between PBB and thyroid hormones was dependent on age at exposure. Among people exposed before age 16 (*N* = 446), higher PBB exposure was associated with higher total T_3_ (p = 0.01) and free T_3_ (*p* = 0.0003), lower free T_4_ (*p* = 0.04), and higher free T_3_: free T_4_ ratio (p = 0.0001). No significant associations were found among participants who were exposed after age 16. No significant associations were found between TSH and PBB or PCB in any of the analyses conducted.

**Conclusions:**

This suggests that both PBB and PCB are associated with thyroid function, particularly among those who were exposed as children or prenatally.

**Electronic supplementary material:**

The online version of this article (10.1186/s12940-019-0509-z) contains supplementary material, which is available to authorized users.

## Background

Exposure to endocrine-disrupting compounds (EDCs) is prevalent in the modern world. EDCs are present in most plasticizers, pesticides, personal care products, flame retardants, and electronic waste [1–3]. Increased exposures have been associated with the development of cancer, reproductive problems, and hormone dysfunction [4, 5]. Especially troubling, children are believed to be particularly susceptible to the effects of EDCs, with children exposed to EDCs in utero or early in life being at increased risk of problems with reproduction, pubertal development, neurodevelopment, and obesity [6]. However, many of the studies do not have a clear dose-response relationship between higher EDC exposure and health problems, and different studies have inconsistent results.

In 1973, millions of Michigan residents were exposed to polybrominated biphenyl (PBB), a brominated flame retardant and an EDC, when a factory accident caused it to be added to the food supply [7, 8]. The Michigan Chemical Company (owned by the Velsicol Chemical Company) mistakenly shipped approximately 500–1000 pounds of PBB to the Farm Bureau Services, where it was added to livestock feed [7, 8]. Animals across the state were exposed when they ate the contaminated feed. During the 10-month period preceding this discovery and the subsequent quarantine of affected farms, Michigan residents were exposed by eating contaminated meat and dairy [7, 8]. In the aftermath, the people who were believed to have the highest direct exposure – people living on or obtaining food from quarantined farms and the Michigan Chemical Company’s workers and their families – were recruited to investigate the long-term health effects of PBB exposure. These participants, their children, and other members of the community have been followed for the past 40 years as part of the Michigan PBB Registry and have had their current serum levels of PBB regularly assessed, as well as their exposure to the structurally-related polychlorinated biphenyl (PCB), which they were continuously exposed to from typical environmental sources [7–9].

A majority of participants of the Michigan PBB Registry still have detectable PBB levels 40 years after the incident because PBB is lipophilic and biologically persistent [10, 11]. Additionally, PBBs are transferred across the placenta and are present in the breast milk of exposed women, meaning that their children, even those born decades after the incident, can be exposed to PBB during development [12]. People exposed to PBB in utero or as children have been shown to have an increased risk of endocrine-related health conditions, and may experience unique problems compared to those exposed as adults [9]. For example, women exposed to PBB in utero were found to be at increased risk for earlier menarche and spontaneous abortions, while women exposed as adults were not found to be at increased risk [13–15]. Further, women exposed in childhood were at an increased risk for having children with lower Apgar scores [16]. Men exposed in utero reported more genitourinary conditions and slower growth and pubertal development [17, 18].

PBB and PCB may also disrupt thyroid hormone signaling and lead to thyroid disease given the structurally-similarities between these EDCs and thyroid hormones [4]. Proper thyroid hormone signaling is important for normal development, homeostasis, and cell proliferation [19]. The primary thyroid hormone in the blood is thyroxine (T_4_), which can either be bound to carrier proteins and inactive, or free, biologically-active molecules. All cells in the body are targets for thyroid hormones, making thyroid function particularly important for metabolism and fetal development [20, 21]. Free T_4_ is converted to triiodothyronine (T_3_) by deiodinases in target tissues, where it can then bind to thyroid hormone receptors. Receptors bound to T_3_ will then act as transcription factors and cause conformational changes that recruit transcription coactivators to DNA and initiate gene transcription. Smaller amounts of T_3_ (both bound and free) are also present in the blood stream, as is thyroid stimulating hormone (TSH), which regulates T_3_ and T_4_ production and iodine uptake in the thyroid. Thyroid disease can cause further issues with metabolism and weight, fertility, and attentiveness, while subclinical thyroid function may lead to issues with fertility, fetal development, and increase the risk for cardiovascular disease [22–27].

In animals, exposure to PBBs lowered levels of total T_3_ and T_4_ and increased thyroid weight (indicative of thyroid conditions like goiters or thyroiditis), and cell lines exposed to PBB had decreased thyroid hormone mediated gene transcription and decreased dendrite branching [28, 29]. Similarly, studies have shown that increased exposure to PCB associates with thyroid dysfunction, and PCBs can bind to thyroid hormone receptors in rat cell lines [30, 31]. In the developing brains of fetal rats, maternal exposure to PCBs mimics thyroid hormones by driving gene expression and altering cellular composition [32–34]. Studies in humans exposed to PBBs have been less consistent, with some studies finding no association between increased PBB exposure and increased risk for thyroid disease, and others finding increased risk for thyroid disease both in highly-exposed chemical workers and in women [35–37].

This study investigates whether increased PBB and PCB exposure associates with thyroid hormone levels in members of the Michigan PBB Registry. This is the largest study of thyroid hormone levels and PBB. Because PBB exposure (unlike PCB exposure) in this cohort occurred during a single, unique time-point, we can also estimate participants’ age when exposed to PBB, and test whether their age at exposure to PBB and current PBB level predict thyroid function. Additionally, because many of the health effects associated with PBB were in those who were exposed when they were young [9, 14, 15, 17, 18], we can then test whether the association between PBB exposure and thyroid function differs in people exposed before age 16 compared to those exposed after age 16.

## Methods

### Participant selection

Participants were selected from the Michigan PBB Registry. Recruitment of these participants has been described in detail elsewhere [37]. Briefly, the Michigan PBB Registry was originally started after the agricultural accident by the Michigan Department of Community Health (MDCH), and recruited individuals believed to have the highest exposure to PBB: people living on quarantined farms, people who ate food from quarantined farms, and chemical workers and their families. Biological samples and health information from the original registry participants, their children, and other member of the community who were exposed to PBBs are still being collected and added to the registry (http://pbbregistry.emory.edu/).

For this study, 744 blood samples were selected that had exposure to PBB and PCB already assessed and had remaining serum for hormone analyses. These samples came from 717 participants and were collected between 2004 and 2015. For those with multiple samples over time, the most recent sample was used for each participant. PBB level, PCB level, lipid level, and thyroid hormone level were all analyzed from the same sample. Of those with serum samples, 596 of these participants had completed a health questionnaire, which was used to determine whether they were currently on thyroid medication. Two participants reported being on thyroid medication at the time of their blood draw and were removed from all analyses (*N* = 715). Ten additional participants reported having a thyroid condition, but did not report taking medication. A sensitivity analysis was conducted with just the 584 participants to ensure that the results held in the participants with known thyroid medication status.

### Exposure measurement

The Michigan PBB Registry has been assessed for both PBB and PCB. There are 209 possible congeners of PBB and PCB that exist based on the number and position of the halogen molecules around the biphenyl rings [38]. The primary congener in the technical mixture that contaminated the food supply in the 1970’s was PBB-153 [7, 8, 38]. Exposure to four congeners of PBB (PBB-153, PBB-101, PBB-77, and PBB-180) and four congeners of PCB (PCB-153, PCB-180, PCB-138, PCB-118) was previously assessed in members of this registry using gas chromatography-tandem mass spectrometry [39]. The limit of detection (LOD) was 2 pg/mL for PBB-153; 4.5 pg/mL for PBB-77; 3.9 pg/mL for PBB-101; 5.6 pg/mL for PBB-180; 0.7 pg/mL for PCB-180; 1.6 pg/mL for PCB-153; 1.2 pg/mL for PCB-138; and 1.4 pg/mL for PCB-118. The extraction recovery ranged from 83.2–99.2%. The accuracy ranged from 89 to 119%, and the precision ranged from 2.8–8.5%. The value for any congener below the LOD in a sample was imputed as the LOD divided by the square root of 2 [40]. The congeners were summed to give a total PBB value and total PCB value per person. The congeners of PBB and PCB were variably correlated with each other with the PBB congeners being more correlated to each other than to the PCB congeners (Additional file 1: Figure S1).

### Lipid measurement

A Triglyceride Quantification Assay Kit (Abnova Corporation) was used to measure the total serum triglyceride content, and a Cholesterol Assay Kit (Caymen Chemical Company) was used to measure total serum cholesterol content. Both were done according to manufacturer’s instructions. Total lipid amount was calculated based on these components as described elsewhere [41, 42].

### Thyroid hormone measurement

Thyroid hormone levels (total and free T_4_, total and free T_3_, and TSH) were analyzed from either serum or plasma with a Beckman Coulter Access II chemiluminescent immunoassay (Beckman Coulter) by the Emory Clinical Translational Research Laboratory. All analyses were conducted per manufacturer’s instructions, as previously described [37]. Technical replicates were run throughout the experiment and were highly correlated with each other (mean correlation = 0.99). Hormone levels were measured and detectable for all the samples, except for one sample that did not have sufficient quantity for analyzing the total T_4_ level. Population-based clinical ranges for this assay were used to determine whether any of the samples had thyroid disease (TSH: 0.34–5.60 *μ* IU/mL; free T_4_: 0.61–1.12 ng/dL; free T_3_: 2.5–3.9 pg/mL; total T_4_ in women: 5.0–9.80 *μ* g/dL; total T_4_ in men: 6.1–12.2 *μ* g/dL; total T_3_: 0.87–1.78 ng/mL).

### Statistical analyses

All exposure and hormone values were transformed using a natural log so that they would be less skewed. A ratio between free T_3_ and free T_4_ was calculated, and since it was already not skewed, it was not transformed. Associations between thyroid hormone levels (as the dependent variable) and exposure to PBB (as the independent variable) were assessed using linear regression models. All models included covariates for age, sex, total PCB level, and total lipid amount. Lipids were adjusted for as a covariate instead of on a wet weight basis to allow for more flexibility and limit bias [43]. Additionally, the cohort was subset into whether they had high exposure to PBB, high exposure to PCB, high exposure to both, or high exposure to neither (based on median split). The same models were tested in these subsets separately. We tested for interaction between age of exposure to PBB and level of exposure by adding an interaction term (age of exposure × PBB level) to the model. The cohort was also subset into those who were exposed before finishing puberty (defined as their age in 1973 being less than or equal to 16) and those who were exposed after finishing puberty (defined as their age in 1973 being older than 16). Sixteen was chosen as the cut-point in order to be consistent with early studies done in children exposed to PBB [44], more recent studies in this study population [10, 11, 45], and the approximate age when puberty is complete in the United States [46]. However, to ensure that the associations seen were not due to arbitrary stratification of the data, the cohort was subset into quartiles of age of exposure and by the median age of exposure. In the analyses in these subsets, age, sex, total PCB level, and lipids were covariates. Interaction between PCB and age of exposure to PCB was not tested because PCB exposure in this cohort was continuous and not during a defined agricultural accident, making us unable to estimate when people were first exposed to PCB. We also tested for interaction between sex and level of exposure by adding an interaction term (sex × exposure level) to the model. The models were also subset by sex. In the analyses in these subsets, age, total PCB level, and lipids were covariates. An alpha level of 0.05 was used as the cut-off for statistical significance in all analyses.

## Results

Participants of this study (*N* = 715) were highly exposed to PBB (range: 0.01–236.73 ppb; Table 1), with 92% having PBB levels higher than the median for a representative sample of the United States [47] and exposed to PCB (range: 0.03–8.12 ppb), at levels similar to typical exposure for people in the United States [3]. There were more female than male participants, and the average age at time of blood draw was 51 years old (range: 18–88 years). Because participants were largely exposed to PBB during a factory accident during the 1970’s, age at exposure is correlated with their current age (r = 0.96; *p* <  2.2e-16). Older participants had higher levels of PBB and PCB (PBB: r = 0.48, p <  2.2e-16; PCB: r = 0.59, p <  2.2e-16), and men had higher PBB and PCB levels than women (PBB: *p* = 0.01, PCB: *p* = 0.0007). This pattern was consistent when the cohort is stratified by those who were exposed before finishing puberty (age 16) versus those who were exposed after finishing puberty.

**Table 1:** Cohort Demographics

	Total Cohort (*N* = 715)	Exposed before puberty^d^ (*N* = 446)	Exposed after puberty^e^ (*N* = 269)	*P*-value
Current age^a^ (years)	51.19 ± 15.21	41.62 ± 9.53	67.07 ± 7.73	< 2.2e-16
Age exposed^a^ (years)	13.60 ± 12.21	5.47 ± 5.19	27.08 ± 7.78	< 2.2e-16
Number male^b^	275 (38.4%)	120 (26.9%)	155 (57.6%)	6.28e-16
Total PBB (ppb)^c^	0.34 (5.79)	0.22 (5.73)	0.72 (4.48)	< 2.2e-16
Total PBB (ng/g lipid)^c^	51.11 (6.11)	31.84 (5.95)	111.99 (4.80)	8.39e-3
Total PCB (ppb)^c^	0.65 (2.76)	0.45 (2.64)	1.21 (2.13)	< 2.2e-16
Total PCB (ng/g lipid)^c^	98.88 (2.83)	66.81 (2.63)	189.43 (2.24)	< 2.2e-16

^a^Mean and standard deviation

^b^Frequency and percentage

^c^Geometric mean and geometric standard error

^d^Exposed before finishing puberty (Age of exposure <= 16)

^**e**^Exposed after finishing puberty (Age of exposure > 16)

**Fig. 1:**
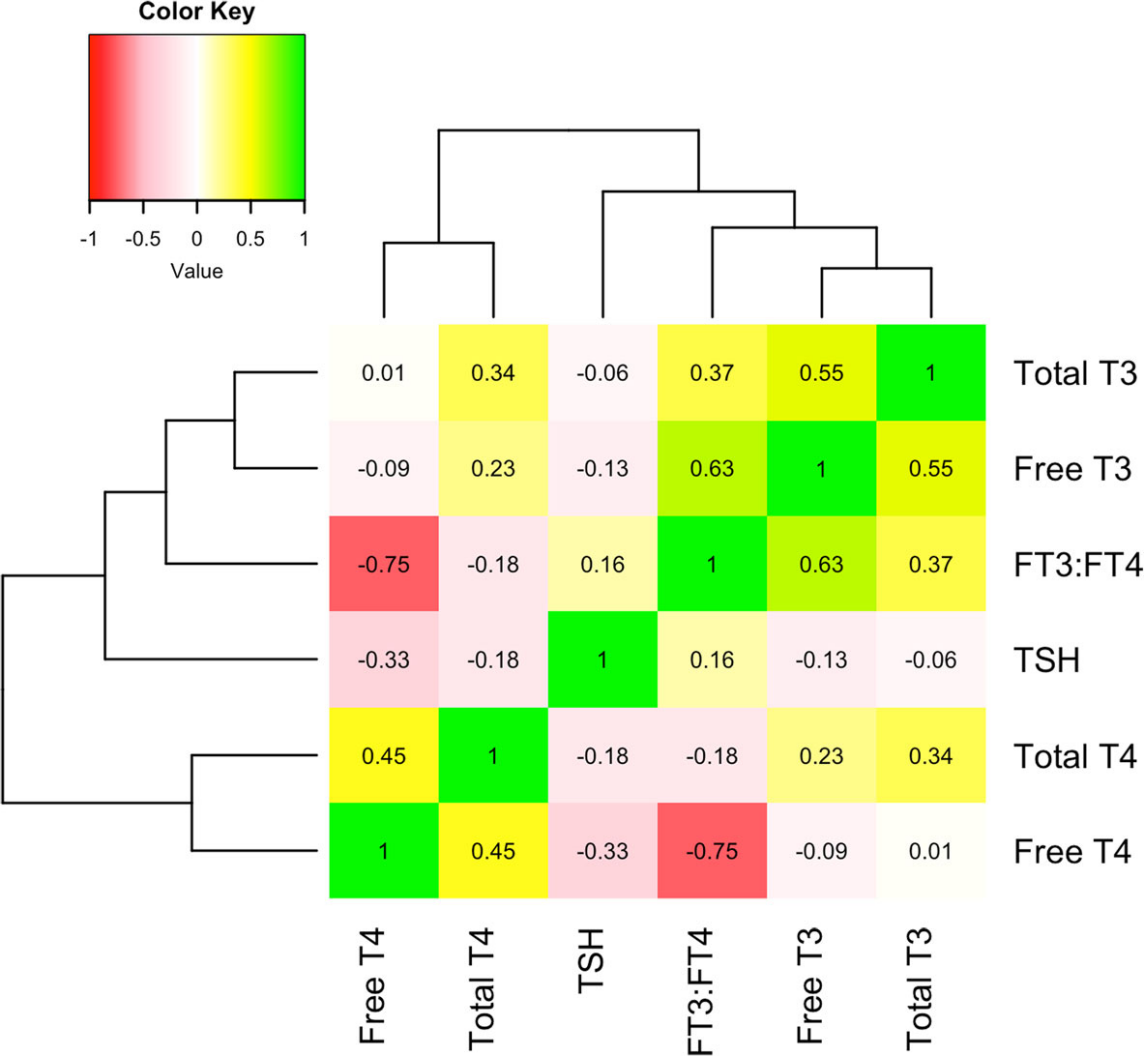
Correlation of thyroid hormone levels. The different thyroid hormone levels measured in this cohort were correlated with each other, and clustered so that the most correlated hormone levels are together (numbers are Pearson’s correlation coefficient). TSH was negatively correlated with the rest of the thyroid hormones, as expected since it is negatively regulated by them, and positively correlated with the free T_3_: free T_4_ ratio. Total and free T_4_ were moderately correlated, as were total and free T_3_. Total T_4_ was positively correlated with both total and free T_3_. Free T_4_ had a weak correlation with both total and free T_3_. The free T_3_: free T_4_ ratio is positively associated with total and free T_3,_ but was negatively associated with total and free T_4_ (as expected). All correlations were statistically significant except for the association of total T_3_ with free T_4_ and TSH (*p* < 0.05)

As expected, most of the thyroid hormone levels were significantly correlated with each other (Fig. 1). For each thyroid assay, a majority of samples were within the usual, population-based range (Additional file 1: Figure S2). A few participants (*N* = 5) had thyroid hormone levels indicative of hypothyroidism (defined as having either a total or free T_4_ level lower than the population-based range and having a TSH level higher than the population-based range), and 7 participants had thyroid hormone levels indicative of hyperthyroidism (defined as having either a total or free T_4_ level higher than the population-based range and having a TSH level lower than the population-based range). Total T_3_, free T_3,_ and free T_3_: free T_4_ ratio were negatively associated with age (r = − 0.30, *p* <  2.2e-16; r = − 0.18, *p* = 5.68e-7; and r = − 0.20, *p* = 3.41e-8, respectively), and free T_4_ was positively associated with age (r = 0.09, *p* = 0.01). This is reflected in the different means for those three hormones in the group exposed after finishing puberty (age 16) and the group exposed before finishing puberty (Table 2). Thyroid hormone levels were also associated with sex, with men having lower levels of total T_3_ (*p* = 0.001) and total T_4_ (*p* = 0.02), and higher levels of free T_3_ (*p* = 0.03).

**Table 2:** Thyroid hormone levels in Michigan PBB Registry

	Total Cohort	Exposed before puberty^c^	Exposed after puberty^d^	*P*-value
Total T_4_^a^ (*μ* g/dL)	9.06 (1.20)	9.02 (1.20)	9.12 (1.21)	0.46
Total T_3_^a^ (ng/mL)	1.01 (1.26)	1.06 (1.25)	0.94 (1.24)	1.64e-10
Free T_4_^a^ (ng/dL)	0.78 (1.19)	0.77 (1.18)	0.80 (1.21)	5.09e-03
Free T_3_^a^ (pg/mL)	3.16 (1.16)	3.22 (1.16)	3.07 (1.15)	1.98e-05
TSH ^a^ (*μ* IU/mL)	1.60 (1.97)	1.55 (1.93)	1.67 (2.03)	0.15
Free T_3_: Free T_4_^b^	4.12 ± 0.90	4.26 ± 0.91	3.90 ± 0.85	1.41e-07

^a^Geometric mean and geometric standard error

^b^Mean and standard deviation

^c^Exposed to PBB before finishing puberty (Age of exposure <= 16)

^**d**^Exposed to PBB after finishing puberty (Age of exposure > 16)

**Fig. 2:**
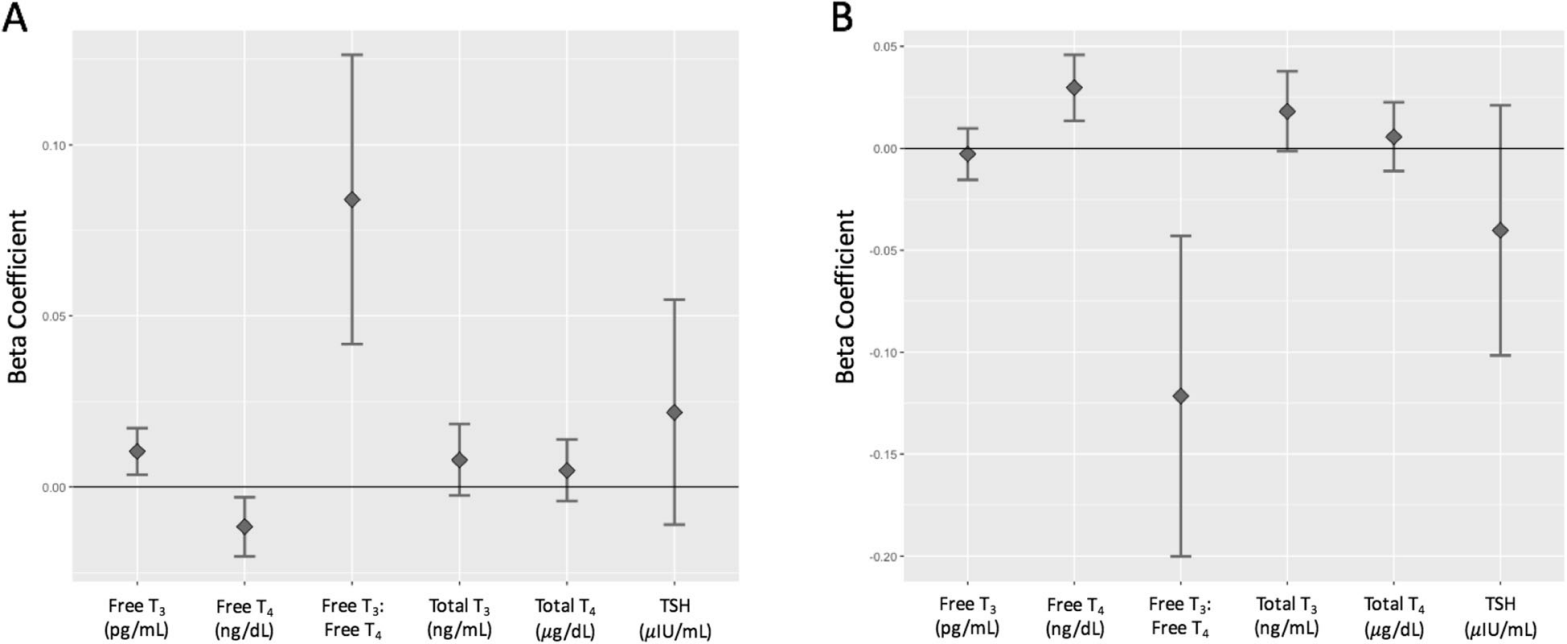
Association of PBB exposure and thyroid hormone levels. The beta coefficients and 95% confidence interval (y-axis) for total PBB level (**a**) and total PCB level (**b**) from the regression of the five thyroid hormone levels (x-axis), also controlling for age, sex, and lipids. Free T_3_ (*p* = 0.002), free T_4_ (*p* = 0.008), and the free T_3_: free T_4_ (*p* = 0.0001) ratio are significantly associated with total PBB exposure. Free T_4_ (*p* = 0.0002) and the free T_3_: free T_4_ ratio (p = 0.002) are significantly associated with total PCB exposure

**Fig. 3:**
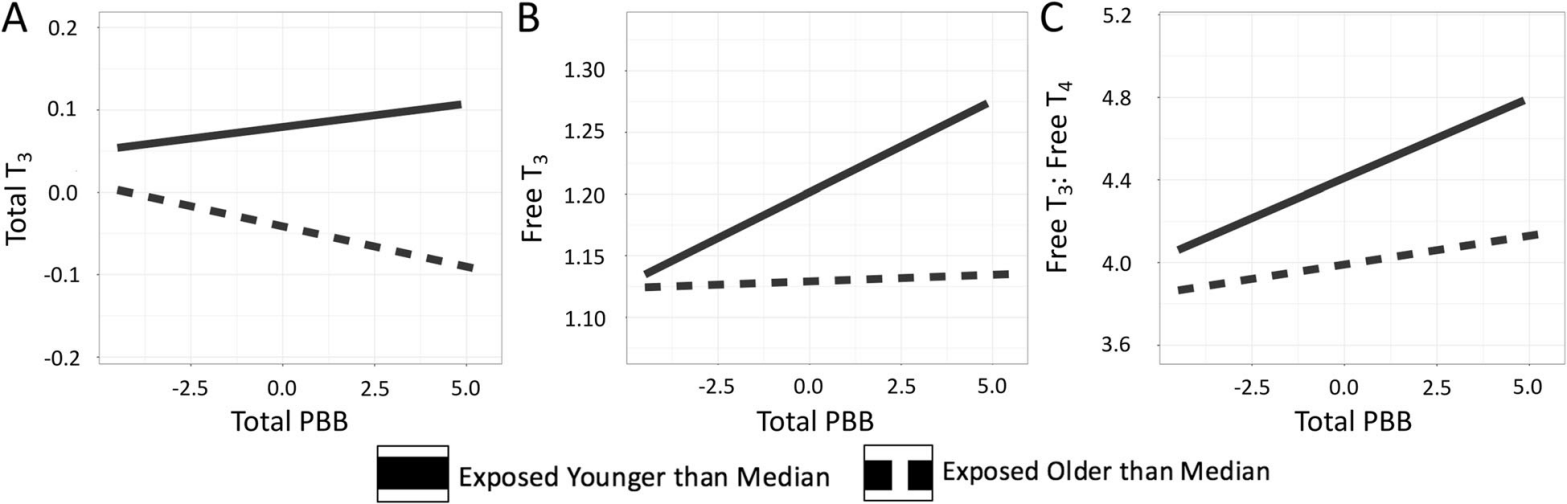
Age of exposure interacts with PBB level to predict thyroid hormone levels. There was a significant interaction between age of exposure and total PBB level in predicting total T_3_ (*p* = 0.03) (**a**), free T_3_ (p = 0.002) (**b**), and the free T_3_: free T_4_ ratio (*p* = 0.01) (**c**). The interaction term was not significant for predicting total T_4_, free T_4_, and TSH. The interaction term in the model was built with two continuous variables, but in order to visualize the interaction, age of exposure was dichotomized around the median and plotted with PBB level on the x-axis and hormone level on the y-axis

In the total cohort, PBB levels were positively associated with free T_3_ levels (t = 3.01, *p* = 0.002), free T_3_: free T_4_ ratio (t = 3.90, *p* = 0.0001), and negatively associated with free T_4_ (t = − 2.64, *p* = 0.008). PBB levels were not associated with total T_4_ (t = 1.05, *p* = 0.29), total T_3_ (t = 1.48, *p* = 0.13), or TSH (t = 1.30, *p* = 0.19) (Fig. 2a, Table 3). PCB, on the other hand, was positively associated with free T_4_ (t = 3.63, *p* = 0.0002) and negatively associated with the free T_3_: free T_4_ ratio (t = − 3.03, *p* = 0.002). PCB was not associated with total T_4_ (t = 0.66, *p* = 0.50), total T_3_ (t = 1.82, *p* = 0.06), free T_3_ levels (t = − 0.42, *p* = 0.66), or TSH (t = − 1.28, p = 0.19) (Fig. 2b, Table 3). These associations were consistent when conducted in the subset of participants that reported their thyroid medication status (Additional file 1: Table S1). Additionally, the direction of the effect of PBB and PCB on thyroid hormone levels was consistent if the population was subset by exposure level to the two chemicals (Additional file 1: Tables S2-S5). There was no statistically significant interaction between gender and PBB to predict any of the thyroid measures (Additional file 1: Table S6), but there was some evidence of an interaction between gender and PCB exposure level to predict free T_4_ and free T_3_: free T_4_ ratio, with there only being an association between PCB and thyroid hormone levels in women (Additional file 1: Table S7). An interaction between age of exposure to PBB and total PBB level was significant for predicting total T_3_ (t = − 2.15, *p* = 0.03), free T_3_ (t = − 3.07, p = 0.002), and the free T_3_: free T_4_ ratio (t = − 2.39, *p* = 0.01; Fig. 3). The interaction term was not significant for predicting total T_4_ (t = − 0.84, *p* = 0.39), free T_4_ (t = 0.68, *p* = 0.49), and TSH (t = − 0.15, *p* = 0.87).

**Table 3:** Regression coefficients from the association of PBB exposure and thyroid hormone levels in total study population

		Total T_4_ (*μ* g/dL)	Total T_3_ (ng/mL)	Free T_4_ (ng/dL)	Free T_3_ (pg/mL)	TSH (*μ* IU/mL)	Free T_3_: Free T_4_ ratio
Model	Variables	*β* (95% CI)	*β* (95% CI)	*β* (95% CI)	*β* (95% CI)	*β* (95% CI)	*β* (95% CI)
1	Total PBB (ppb)	0.0048 (− 0.0041, 0.0139)	0.0079 (− 0.0025, 0.0184)	− 0.0116(− 0.0202, − 0.0030)	0.0104 (0.0036, 0.0172)	0.0218 (− 0.0110,0.0547)	0.0840 (0.0418, 0.1262)
	Current Age (years)	− 0.0006 (− 0.0013, 0.0012)	−0.0056 (− 0.0071, − 0.0042)	0.0005 (− 0.0006, 0.0017)	− 0.0028 (− 0.0038, − 0.0019)	0.0032 (− 0.0013,0.0078)	−0.0141 (− 0.0200, − 0.0082)
	Sex	−0.0449 (− 0.0763, − 0.0136)	−0.0154 (− 0.0515, 0.0212)	−0.0004 (− 0.0304, 0.0294)	0.0414 (0.0178, 0.0651)	0.0311 (− 0.0830, 0.1452)	0.1555 (0.0091, 0.3020)
	Lipids (mg/dL)	−0.00008 (− 0.0001, − 0.00001)	−0.0001 (− 0.0002, − 0.0001)	−0.00009 (− 0.0001, − 0.00002)	−0.0001 (− 0.0001, − 0.00009)	0.0002 (− 0.00001, 0.0004)	−0.0001 (− 0.0004, 0.0001)
	Total PCB (ppb)	0.0057 (−0.0111, 0.0225)	0.0181 (− 0.0013, 0.0377)	0.0297 (0.0136, 0.0458)	− 0.0027 (− 0.0154, 0.0099)	−0.0402 (− 0.1015, 0.0210)	−0.1215 (− 0.2001, − 0.0429)
2	Total PBB (ppb)	0.0080 (− 0.0036, 0.0197)	0.0173 (0.0038, 0.0308)	−0.0141 (− 0.0252, − 0.0029)	0.0190 (0.0103, 0.0278)	0.0240 (− 0.0184, 0.0665)	0.1257 (0.0715, 0.1800)
	Current Age (years)	− 0.00027 (− 0.0016, 0.0010)	−0.0063 (− 0.0079, − 0.0047)	0.0006 (− 0.0006, 0.0019)	−0.0034 (− 0.0044, − 0.0024)	0.0031 (− 0.0018, 0.0081)	−0.0170 (− 0.0233, − 0.0106)
	Sex	−0.0434 (− 0.0749, − 0.0118)	−0.0105 (− 0.0471, 0.0260)	−0.0016 (− 0.0318, 0.0284)	0.0457 (0.0220, 0.0693)	0.0321 (− 0.0828, 0.1471)	0.1759 (0.0290, 0.3228)
	Lipids (mg/dL)	−0.00008 (− 0.0001, − 0.00001)	−0.0001 (− 0.0002, − 0.0001)	−0.00009 (− 0.0001, − 0.00002)	−0.0001 (− 0.0001, − 0.00009)	0.0002 (− 0.00001, 0.0004)	−0.0001 (− 0.0004, 0.0001)
	Total PCB (ppb)	0.0055 (−0.0112, 0.0224)	0.0177 (− 0.0017, 0.0372)	0.0298 (0.0138, 0.0459)	− 0.0031 (− 0.0157, 0.0094)	−0.0403 (− 0.0101, 0.0210)	−0.1234 (− 0.2018, − 0.0451)
	Age exposed × Total PBB	−0.0002 (− 0.0009, 0.0003)	−0.0008 (− 0.0016, − 0.00007)	0.0002 (− 0.0004, 0.0008)	−0.0007 (− 0.0012, − 0.0002)	−0.0001 (− 0.0026, 0.0022)	−0.0038 (− 0.0069, − 0.0006)

**Fig. 4:**
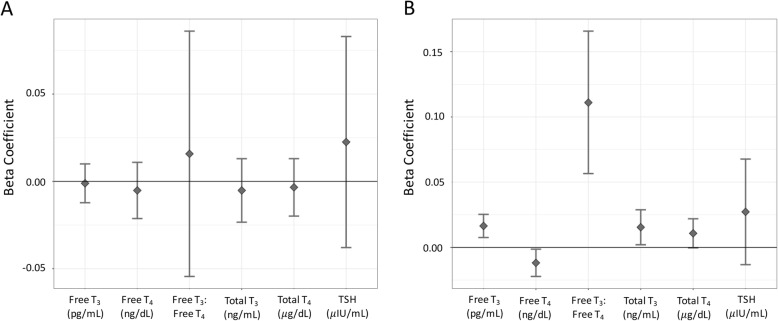
Association of PBB exposure and thyroid hormone levels stratified by exposure before or after finishing puberty. The total cohort was stratified into people who were either exposed to PBB after finishing puberty (**a**) or before finishing puberty (**b**) and the association between total PBB and all six thyroid hormone measures was tested, controlling for age, total PCB level, sex, and lipid levels. PBB and thyroid hormone levels were not associated in the subset exposed after finishing puberty, but in the subset exposed before finishing puberty, PBB and total T_3_ (*p* = 0.02), free T_3_ (*p* = 0.0003), free T_4_ (p = 0.02), and the free T_3_: free T_4_ ratio (*p* = 7.40e-5) were associated

Because there was a significant interaction between exposure and age when exposed, the association between PBB level and thyroid hormone levels was examined separately among participants exposed after finishing puberty (age 16) and participants exposed before finishing puberty. In the people that were exposed after finishing puberty, there were no associations between PBB level and any of the thyroid hormones (total T_4_: t = − 0.40, *p* = 0.68; total T_3_: t = − 0.56, *p* = 0.57; free T_4_: t = − 0.63, *p* = 0.52; free T_3_: t = − 0.19, *p* = 0.84; TSH: t = 0.73, *p* = 0.46, free T_3_: free T_4_ ratio: t = 0.44, *p* = 0.65; Fig. 4a). However, among the people who were exposed before finishing puberty, higher PBB was associated with an increase in total T_3_ (t = 2.25, *p* = 0.02), free T_3_ (t = 3.63, *p* = 0.0003), and free T_3_: free T_4_ ratio (t = 4.00, *p* = 7.40e-5), and a decrease in free T_4_ (t = − 2.22, p = 0.02). PBB was not associated with either total T_4_ (t = 1.89, *p* = 0.05) or TSH (t = 1.32 *p* = 0.18) in people exposed before finishing puberty (Fig. 4b). If instead the sample was stratified by quartile (Additional file 1: Table S8) or median age of exposure (Additional file 1: Table S9), the associations between PBB and thyroid hormone levels were found in the people in the younger groups compared to the oldest group.

## Discussion

This cross-sectional study leveraged data from 715 people who had high exposure to PBB due to a factory accident over 40 years ago. Among these participants, there was evidence that PBB and PCB disrupted thyroid hormones, with higher PBB exposure associating with higher free T_3_ and free T_3_: free T_4_ ratio, and decreased free T_4_ levels, and PCB being associated with higher free T_4_ levels and lower free T_3_: free T_4_ ratio. Previous research demonstrated that people exposed to PBBs report higher frequency of thyroid disease (particularly hypothyroidism) [36, 37]. However, in this study, PBBs only associated with lower free T_4_ and not with higher TSH (when clinical diagnoses of hypothyroidism require a low T_4_ level and a high TSH level), and with an increase of T_3_ but not a decrease in TSH (when clinical diagnoses of hyperthyroidism require a high T_3_ or high T_4_ level and a low TSH level) [36, 37, 48–50]. This study’s findings are consistent with the previous study in a rat model, which reported a decrease in T_4_. However, that study also found a decrease in T_3_ [28]. It is possible that this difference in response could be due to differences in dose, with rats being fed a minimum of 5 ppm PBB (human equivalent of 160 ppm), where this cohort has a geometric mean exposure of 0.34 ppb [9, 28]. The negative association between PBB and free T_4_ is also inconsistent with both the published positive association between PCB and free T_4_ [37] and the positive association between PCB and free T_4_ found in this study. It is not clear why there is a difference in association between free T_4_ and PBB or PCB, since the animal study showed a negative association with both PBB and PCB and free T_4_ [28]. However, other studies have found a negative association between PCB and T_4_, consistent with the PBB results [51]. More research is therefore needed to determine whether there are factors, such as dose or the precise mixture of congeners of PCB, that change how PCB and thyroid hormones associate [52].

Even though the associations between thyroid hormone levels and PBB in this study are not entirely consistent with a clinical outcome of thyroid disease, variations in thyroid hormone levels within their normal ranges can still impact health. Variations in thyroid hormone levels within their normal ranges are associated with increased triglycerides, cholesterol levels, BMI, and cardiovascular problems [53, 54]. For men, higher free T_3_ levels is associated with gynecomastia (enlarged breasts) [55]. For women, subclinical hypothyroidism is associated with adverse pregnancy outcomes, like miscarriage, pre-eclampsia, and subfertility that is unexplained by other sources [56]. Therefore, people with higher exposure to PBBs may be at greater risk for metabolic and reproductive problems even if their thyroid hormone levels are still within the usual population range. This was especially true among the people exposed as children, who also had higher total T_3_, free T_3_, and free T_3_: free T_4_ ratio and lower free T_4_ with higher exposure to PBB.

This study found stronger associations between PBB and thyroid hormone levels in people who were exposed younger. This is consistent with previous research in this cohort which has found spontaneous miscarriage, offspring with lower Apgar score, and earlier age of menarche in women exposed younger, and genitourinary problems and slower pubertal development in men exposed younger [14–18]. Additionally, it is consistent with studies that have found that developmental stages are particularly vulnerable to disruption from environment contaminants [57, 58]. Finding stronger associations between PBB exposure and variations in thyroid hormone levels in people exposed younger is of particular concern given that children of women who were exposed can still be exposed to PBBs through placental transfer and breastfeeding. It should be noted that this association was seen in the youngest subset of the study population, regardless of whether the population was divided into quartiles, by median, or by age 16. Therefore, younger age of exposure is a risk, but other studies would be needed in order to determine the precise window of vulnerability.

Previous work on the association between PBB and thyroid function in the Michigan PBB Registry has had conflicting results. Some studies found an association between level of PBB exposure and hypothyroidism [36, 37], while others did not demonstrate the same association between PBB and thyroid disease [35]. In the only other study of the association between thyroid hormone levels and PBB exposure level, there was evidence of association between PBB and free T_3_ levels, but not with other thyroid hormones [37]. However, the current study had almost 30% more participants (*N* = 715 vs. *N* = 551), and the effect sizes in the total cohort from this study are comparable, but more precise, to what was found previously, indicating that this study had more power to detect a statistical association. Including people in this study who reported a thyroid condition but were not taking medication should not have biased the results, because none of the transformed thyroid hormone levels differed significantly between the current study and the previous study which excluded anyone who had ever been diagnosed with a thyroid problem even if they were not treated with thyroid medication. Additionally, similar to the previous study, we found a negative correlation between free T_4_ and PCB, and there was a stronger association between PCB and PBB and thyroid hormone levels in women. However, given the large overlap of samples in both studies, this should not be viewed as a replication of previous results, but rather as further support for those conclusions and a more detailed exploration of the role of age of exposure in increasing PBB-associated health risks.

This builds upon the previous work in this cohort not only by having a larger sample size and stratifying by age at exposure, but also by analyzing the free T_3_: free T_4_ ratio. A higher free T_3_: free T_4_ ratio is associated with several metabolic parameters, including insulin resistance, enlarged waist circumference, higher BMI, and blood glucose levels in people without thyroid conditions [23–25]. A higher free T_3_: free T_4_ ratio is also associated with an increased risk for non-alcoholic fatty liver disease in both in euthyroid and hypothyroid individuals, independently of other metabolic parameters [59]. This is of interest given that, in animal models, high exposure to PBBs results in liver pathologies including increased vacuolation and altered drug metabolism in their livers [60–63]. Given the evidence in animal models and the associations reported in human cohorts, it is possible that people that are highly exposed to PBBs may be at increased risk for a variety of metabolic conditions, however, this has not been directly studied in a human cohort.

One possible explanation for finding an association between higher PBB levels and lower free T_4_ and higher total T_4_ (although the association with total T_4_ was not statistically significant) is that higher PBB exposure is associated with alterations of estrogen levels and an increase in thyroxine-binding globulin (TBG). TBG binds a majority of the T_4_ in blood, rendering it biologically unavailable. One mechanism that leads to increased synthesis of TBG is increased levels of estrogen [64]. While whether higher TBG is associated with lower free T_4_ is still unclear [48, 65], previous research has reported that variations in euthyroid, serum levels of total T_4_ are positively associated with urinary estrogen and progesterone levels, and that free T_4_ is associated with shorter cycle and follicular phase length [66]. If the associations between higher PBB and thyroid hormone levels are due to alterations in estrogen levels resulting in higher TBG, it could indicate that PBB is weakly estrogenic. While there is evidence that PBB can alter hormone metabolism in rats [62, 67, 68], alters menstrual function in monkeys [69], and is associated with earlier pubertal timing in women [15], there is no direct evidence that PBB is estrogenic at this time, and TBG levels were not measured in this cohort.

Interestingly, this study found that PBB is associated with lower free T_4_ and higher free T_3_ and free T_3_: free T_4_ ratio. A majority of thyroid hormone in blood is T_4_, which is then converted to T_3_ at the target tissue, and the levels of both negatively regulate TSH. However, finding opposite effects of PBB on T_3_ and T_4_ could be due to PBB affecting the conversion of T_3_ to T_4_ (for example, by PBB altering deiodinase activity) [70]. However, not enough is known about the mechanism behind how PBB affects thyroid function to fully be able to explain this result. This is not consistent with the findings in animal models, which found extremely high doses of PBB were associated with decreases in T_3_ and T_4_ [28], or with previous studies on PBB and hypothyroidism, which would require both higher TSH and lower T_4_ [36, 37]. Therefore, it is also possible that PBB affects thyroid function differently at lower doses than at high doses (leading to decreases in T_3_ and T_4_ in animal models and increases in TSH in humans), or that higher T_3_ and lower T_4_ is a compensation mechanism for PBB’s other effects on cells. Research in model systems could elucidate the cellular mechanisms underlying the effects of PBB on thyroid function.

This study does have limitations. This is an epidemiological study that only measured circulating thyroid hormone levels, and unfortunately, we were not able to measure other factors, such as thyroid ultrasounds, menstrual function, TBG levels, thyroid antibody levels, or oral contraceptive use in the entire study population. Therefore, it is less clear how PBB may mechanistically interfere with thyroid hormone levels. Additionally, there is a lack of information on possible thyroid autoimmunity (which may impact on how EDCs effect the thyroid). Future studies that investigate the effect of PBB on the thyroid gland itself or whether the immune system is a mediator are warranted, either in different human cohorts or in animal models. Finally, it is not known exactly when these participants were exposed to PCB. Therefore, we were unable to test whether the effects of PCB exposure also depend on when people were exposed.

## Conclusions

This study did find that higher, current PBB and PCB levels were associated with thyroid hormone levels. It builds on previous studies that found an increased risk for thyroid disease in women exposed to PBBs and PCBs by leveraging that this cohort was exposed primarily during a narrow time frame to find that there was only an association between higher PBB and thyroid hormone measures in people who were exposed to PBB before puberty. Our finding suggest that this cohort may still be at risk for metabolic- and endocrine-related conditions even 40 years after they were exposed, and that people who are exposed younger may be more vulnerable to the endocrine-disrupting effects of PBBs.

## Additional file

Additional file 1: **Figure S1.** Correlations of exposure congeners in this cohort. **Figure S2.** Thyroid hormone levels by subject in this cohort. **Table S1.** Association of PBB exposure and thyroid hormone levels in subset with thyroid medication status. **Table S2.** Regression coefficients from the association of PBB exposure and thyroid hormone levels in subset with PBB exposure below the median and PCB exposure below the median (*N* = 216). **Table S3.** Regression coefficients from the association of PBB exposure and thyroid hormone levels in subset with PBB exposure below the median and PCB exposure above the median (*N* = 131). **Table S4.** Regression coefficients from the association of PBB exposure and thyroid hormone levels in subset with PBB exposure above the median and PCB exposure below the median (*N* = 141). **Table S5.** Regression coefficients from the association of PBB exposure and thyroid hormone levels in subset with PBB exposure above the median and PCB exposure above the median (*N* = 227). **Table S6.** Association between PBB and thyroid hormone levels in each gender. **Table S7.** Association between PCB and thyroid hormone levels in each gender. **Table S8.** Regression coefficients from the association of PBB exposure and thyroid hormone levels subset by quartile of age of exposure to PBB. **Table S9.** Regression coefficients from the association of PBB exposure and thyroid hormone levels subset by median of age of exposure to PBB. (DOCX 647 kb)

## Data Availability

The datasets used and/or analyzed during the current study are available from the corresponding author on reasonable request.

## Abbreviations

EDC: Endocrine-disrupting compound
PBB: Polybrominated biphenyl
PCB: Polychlorinated biphenyl
T_3_: Triiodothyronine
T_4_: Thyroxine
TBG: Thyroxine-binding globulin
TSH: Thyroid-stimulating hormone
