# Supplementary material for: Thyroid hormone levels associate with exposure to polychlorinated biphenyls and polybrominated biphenyls in adults exposed as children
Source: Environ Health. 2019 Aug 23;18:75. doi: 10.1186/s12940-019-0509-z (PMC6708149; doi:10.1186/s12940-019-0509-z)
Supplement: Supplementary file 1 — Figure S1. Correlations of exposure congeners in this cohort. Figure S2. Thyroid hormone levels by subject in this cohort. Table S1. Association of PBB exposure and thyroid hormone levels in subset with thyroid medication status. Table S2. Regression coefficients from the association of PBB exposure and thyroid hormone levels in subset with PBB exposure below the median and PCB exposure below the median (N = 216). Table S3. Regression coefficients from the association of PBB exposure and thyroid hormone levels in subset with PBB exposure below the median and PCB exposure above the median (N = 131). Table S4. Regression coefficients from the association of PBB exposure and thyroid hormone levels in subset with PBB exposure above the median and PCB exposure below the median (N = 141). Table S5. Regression coefficients from the association of PBB exposure and thyroid hormone levels in subset with PBB exposure above the median and PCB exposure above the median (N = 227). Table S6. Association between PBB and thyroid hormone levels in each gender. Table S7. Association between PCB and thyroid hormone levels in each gender. Table S8. Regression coefficients from the association of PBB exposure and thyroid hormone levels subset by quartile of age of exposure to PBB. Table S9. Regression coefficients from the association of PBB exposure and thyroid hormone levels subset by median of age of exposure to PBB. (DOCX 647 kb) [file 12940_2019_509_MOESM1_ESM.docx]

**Additional file 1**

**Figure S1: Correlations of exposure congeners in this cohort.** A heat map of the correlation between the level of exposure to each congener in the cohort (unsupervised cluster). While all the congeners of PBB are positively, significantly correlated (p < 0.05), total PBB exposure levels are most correlated to PBB-153 exposure level (the most detected congener) at r = 0.98, and is least correlated with PBB-180 exposure levels (the rarest detected congener) at r = 0.17. All the congeners of PCB are also positively, significantly correlated (p < 0.05), with total PCB exposure levels being most correlated to PCB-153 exposure level at r = 0.99. The correlation between PBB and PCB congeners was more variable r = -0.08-0.31.

**
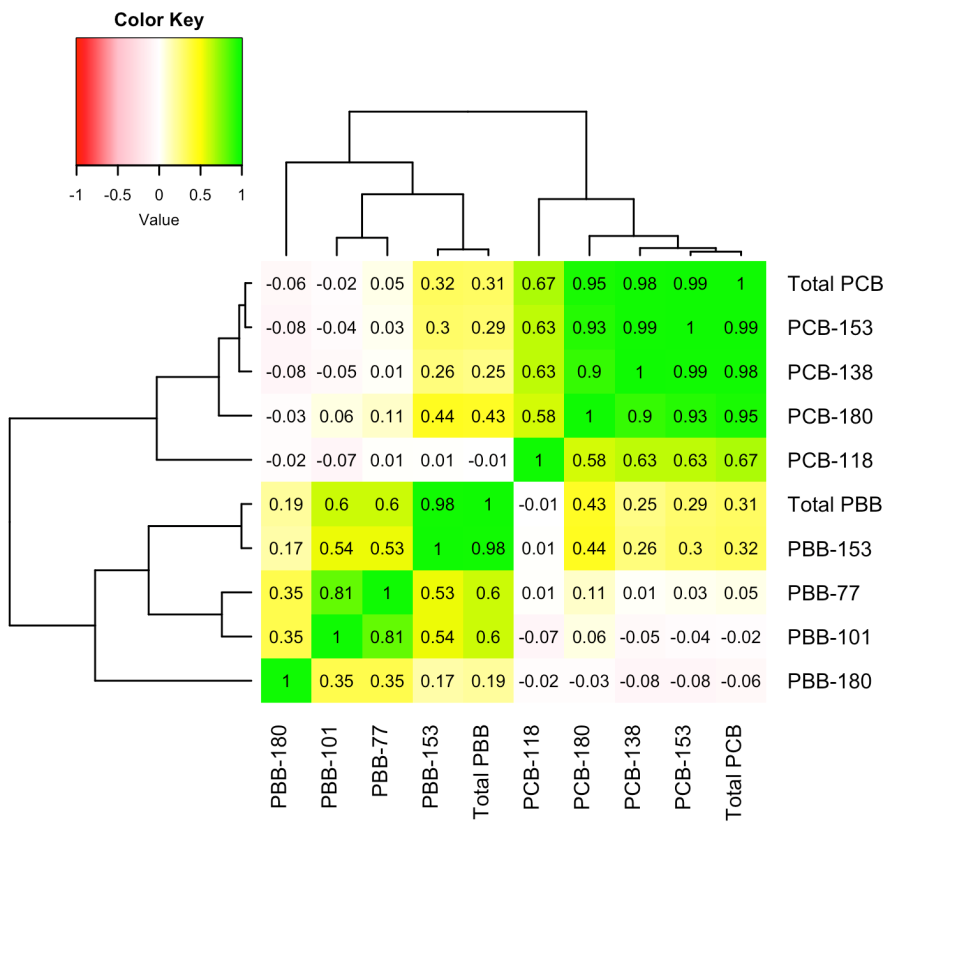
**

**Figure S2:** **Thyroid hormone levels by subject in this cohort.** Below is the graph of thyroid hormone levels measured for each subject. 48.5% of this cohort had all of their thyroid hormones levels within the normal range and 99.8% had at least one of their thyroid hormone levels within the normal range. For total T_4_, 62.1% of female participants were within the normal range (Part A) and 94.2% of male were within the normal range (Part B). For total T_3_, 76.1% of all participants were within the normal range (Part C). For free T_4_, 93.0% of all participants were within the normal range (Part D). For free T_3_, 91.0% of all participants were within the normal range (Part E). For TSH, 96.5% of all participants were within the normal range (Part F). (Black lines = normal range limits).


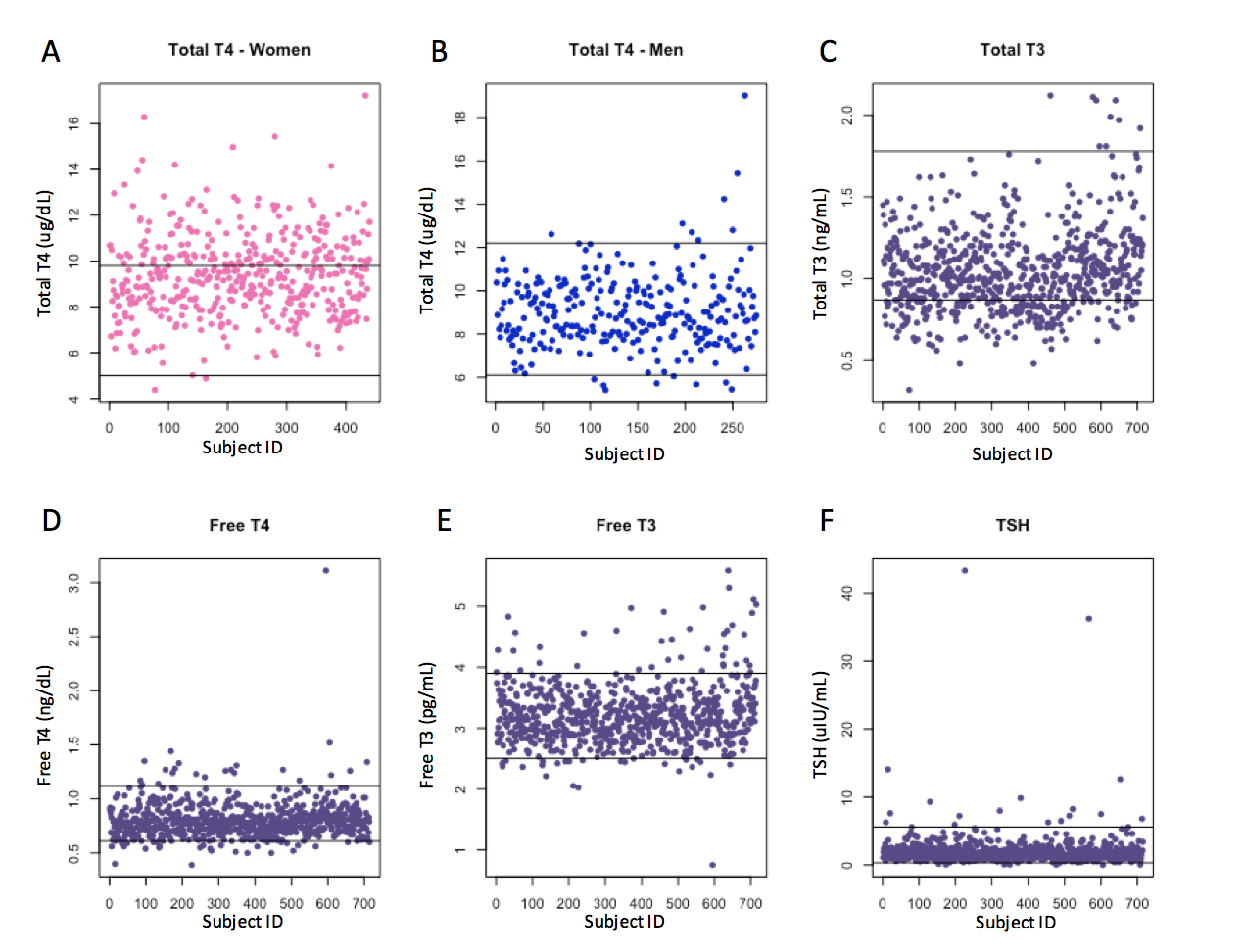


**Table S1: Association of PBB exposure and thyroid hormone levels in subset with thyroid medication status**

|  | Total T_4_ ($\mu$g/dL) | Total T_3_ (ng/mL) | Free T_4_ (ng/dL) | Free T_3_ (pg/mL) | TSH ($\mu$IU/mL) | Free T_3_: Free T_4_ ratio |
| --- | --- | --- | --- | --- | --- | --- |
| Variables | $\beta$  (95% CI) | $\beta$  (95% CI) | $\beta$  (95% CI) | $\beta$  (95% CI) | $\beta$  (95% CI) | $\beta$  (95% CI) |
| Total PBB (ppb) | 0.0048  (-0.0049, 0.0146) | 0.0106  (-0.0011, 0.0225) | -0.0096  (-0.0187, -0.0006) | 0.0130  (0.0053, 0.0207) | 0.0155  (-0.0193,0.0504) | 0.0881  (0.0418, 0.1344) |
| Current Age (years) | -0.0003  (-0.0017, 0.0011) | -0.0053  (-0.0070, -0.0035) | 0.00009  (-0.0012, 0.0014) | -0.0025  (-0.0036, -0.0014) | 0.0023  (-0.0027,0.0074) | -0.0120  (-0.0187, -0.0053) |
| Sex | -0.0454  (-0.0813, -0.0095) | -0.0276  (-0.0709, 0.0155) | -0.0060  (-0.0269, 0.0391) | 0.0229  (-0.0052, 0.0511) | 0.0903  (-0.0375, 0.2182) | 0.0835  (-0.0859, 0.2531) |
| Lipids (mg/dL) | -0.00008  (-0.0001, -0.00001) | -0.0002  (-0.0002, -0.0001) | -0.00009  (-0.0001, -0.00002) | -0.0001  (-0.0001, -0.00008) | 0.0002  (-0.000002, 0.0005) | -0.0001  (-0.0004, 0.0001) |
| Total PCB (ppb) | 0.0068  (-0.0127, 0.0265) | 0.0292  (-0.0056, 0.0528) | 0.0307  (0.0127, 0.0488) | -0.0018  (-0.0172, 0.0135) | -0.0541  (-0.1239, 0.0157) | -0.1197  (-0.2123, -0.0271) |

**Table S2: Regression coefficients from the association of PBB exposure and thyroid hormone levels in subset with PBB exposure below the median and PCB exposure below the median (N =216)**

|  | Total T_4_ ($\mu$g/dL) | Total T_3_ (ng/mL) | Free T_4_ (ng/dL) | Free T_3_ (pg/mL) | TSH ($\mu$IU/mL) | Free T_3_: Free T_4_ ratio |
| --- | --- | --- | --- | --- | --- | --- |
| Variables | $\beta$  (95% CI) | $\beta$  (95% CI) | $\beta$  (95% CI) | $\beta$  (95% CI) | $\beta$  (95% CI) | $\beta$  (95% CI) |
| Total PBB (ppb) | 0.0186  (-0.0091, 0.0463) | 0.0388  (0.0039, 0.0737) | -0.0088  (-0.0340, -0.0163) | 0.0333  (0.0134, 0.0531) | 0.0259  (-0.0666,0.1184) | 0.1808  (0.0506, 0.3110) |
| Current Age (years) | -0.0017  (-0.0044, 0.0009) | -0.0066  (-0.0100, -0.0032) | -0.0002  (-0.0027, 0.0022) | -0.0038  (-0.0057, -0.0018) | 0.0006  (-0.0083,0.0097) | -0.0141  (-0.0268, -0.0014) |
| Sex | -0.0375  (-0.0988, 0.0237) | -0.0162  (-0.0934, 0.0609) | 0.0269  (-0.0287, 0.0826) | 0.0320  (-0.0117, 0.0759) | 0.1110  (-0.0934, 0.3155) | 0.0296  (-0.2582, 0.3175) |
| Lipids (mg/dL) | -0.0001  (-0.0002, -0.00001) | -0.0002  (-0.0003,-0.00005) | -0.0001  (-0.0002, -0.00006) | -0.00009  (-0.0001, -0.000008) | 0.0001  (-0.0003, 0.0005) | -0.0003  (-0.0002, 0.0009) |
| Total PCB (ppb) | 0.0081  (-0.0291, 0.0454) | 0.0415  (-0.0053, 0.0885) | 0.0318  (0.0020, 0.0657) | 0.0011  (-0.0152, 0.0380) | -0.0299  (-0.1543, 0.0943) | -0.0919  (-0.2669, 0.0831) |

**Table S3: Regression coefficients from the association of PBB exposure and thyroid hormone levels in subset with PBB exposure below the median and PCB exposure above the median (N = 131)**

|  | Total T_4_ ($\mu$g/dL) | Total T_3_ (ng/mL) | Free T_4_ (ng/dL) | Free T_3_ (pg/mL) | TSH ($\mu$IU/mL) | Free T_3_: Free T_4_ ratio |
| --- | --- | --- | --- | --- | --- | --- |
| Variables | $\beta$  (95% CI) | $\beta$  (95% CI) | $\beta$  (95% CI) | $\beta$  (95% CI) | $\beta$  (95% CI) | $\beta$  (95% CI) |
| Total PBB (ppb) | -0.0468  (-0.0854,-0.0081) | -0.0506  (-0.1041, 0.0027) | -0.0592  (-0.1046, -0.0138) | 0.0140  (-0.0261, 0.0542) | 0.0782  (-0.0635,0.2200) | 0.2075  (0.0109, 0.4042) |
| Current Age (years) | 0.0036  (0.0010, 0.0062) | -0.0010  (-0.0046, 0.0025) | 0.0015  (-0.0015, 0.0046) | -0.0016  (-0.0044, 0.0010) | 0.0061  (-0.0034,0.0157) | -0.0128  (-0.0261, 0.0004) |
| Sex | -0.0595  (-0.1322, 0.0131) | -0.0599  (-0.1605, 0.0406) | 0.0326  (-0.0527, 0.1180) | 0.0379  (-0.0377, 0.1137) | 0.0618  (-0.2051, 0.3287) | 0.0150  (-0.3551, 0.3852) |
| Lipids (mg/dL) | -0.00009  (-0.0002, 0.00006) | -0.00009  (-0.0003, 0.0001) | -0.00001  (-0.0001, 0.0001) | -0.0001  (-0.0003, -0.00001) | 0.0007  (-0.0001, 0.0013) | -0.0005  (-0.0013, 0.0002) |
| Total PCB (ppb) | -0.0257  (-0.0914, 0.0400) | 0.0295  (-0.1204, 0.0614) | 0.0143  (-0.0629, 0.0915) | 0.0048  (-0.0635, 0.0733) | 0.2216  (-0.0196, 0.4630) | -0.1668  (-0.5015, 0.1678) |

**Table S4: Regression coefficients from the association of PBB exposure and thyroid hormone levels in subset with PBB exposure above the median and PCB exposure below the median (N = 141)**

|  | Total T_4_ ($\mu$g/dL) | Total T_3_ (ng/mL) | Free T_4_ (ng/dL) | Free T_3_ (pg/mL) | TSH ($\mu$IU/mL) | Free T_3_: Free T_4_ ratio |
| --- | --- | --- | --- | --- | --- | --- |
| Variables | $\beta$  (95% CI) | $\beta$  (95% CI) | $\beta$  (95% CI) | $\beta$  (95% CI) | $\beta$  (95% CI) | $\beta$  (95% CI) |
| Total PBB (ppb) | -0.0033  (-0.0210, 0.0143) | 0.0026  (-0.0178, 0.0231) | -0.0060  (-0.0102, 0.0223) | 0.0019  (-0.0111, 0.0151) | 0.0738  (-0.0032,0.1509) | -0.0243  (-0.1234, 0.0748) |
| Current Age (years) | -0.0002  (-0.0051, 0.0010) | -0.0086  (-0.0121, -0.0050) | -0.0014  (-0.0042, 0.0013) | -0.0050  (-0.0073, -0.0027) | 0.0109  (-0.0025,0.0243) | -0.0177  (-0.0350, -0.0005) |
| Sex | -0.0181  (-0.0862, -0.0500) | -0.0117  (-0.0672, 0.0906) | 0.0470  (-0.0156, 0.1097) | 0.0362  (-0.0144, 0.0868) | 0.0422  (-0.2547, 0.3392) | -0.0216  (-0.4032, 0.3599) |
| Lipids (mg/dL) | -0.00006  (-0.0002, -0.00008) | -0.0002  (-0.0004, -0.00008) | -0.00007  (-0.0002, -0.00005) | -0.00008  (-0.0001, 0.00001) | -0.0002  (-0.0008, 0.0003) | 0.00005  (-0.0007, 0.0008) |
| Total PCB (ppb) | 0.0377  (-0.0269, 0.0102) | 0.0227  (-0.0521, 0.0976) | 0.0016  (-0.0578, 0.0611) | 0.0054  (-0.0426, 0.0535) | -0.0690  (-0.3509, 0.2128) | -0.0369  (-0.3999, 0.3252) |

**Table S5: Regression coefficients from the association of PBB exposure and thyroid hormone levels in subset with PBB exposure above the median and PCB exposure above the median (N = 227)**

|  | Total T_4_ ($\mu$g/dL) | Total T_3_ (ng/mL) | Free T_4_ (ng/dL) | Free T_3_ (pg/mL) | TSH ($\mu$IU/mL) | Free T_3_: Free T_4_ ratio |
| --- | --- | --- | --- | --- | --- | --- |
| Variables | $\beta$  (95% CI) | $\beta$  (95% CI) | $\beta$  (95% CI) | $\beta$  (95% CI) | $\beta$  (95% CI) | $\beta$  (95% CI) |
| Total PBB (ppb) | -0.0083  (-0.0354, 0.0188) | -0.0058  (-0.0325, 0.0207) | -0.0270  (-0.0513, -0.0026) | -0.0112  (-0.0283, 0.0058) | 0.0509  (-0.0441,0.1461) | 0.0604  (-0.0484, 0.1694) |
| Current Age (years) | -0.0006  (-0.0032, 0.0019) | -0.0062  (-0.0088, -0.0037) | 0.0022  (-0.00005, 0.0046) | -0.0027  (-0.0043, -0.0010) | -0.0007  (-0.0098,0.0084) | -0.0184  (-0.0289, -0.0080) |
| Sex | -0.0424  (-0.1033, 0.0184) | -0.0210  (-0.0387, 0.0807) | 0.0333  (-0.0879, 0.0211) | 0.0664  (0.0280, 0.1048) | -0.0287  (-0.2422, 0.1847) | 0.3712  (0.1268, 0.6157) |
| Lipids (mg/dL) | -0.00006  (-0.0002, 0.00007) | -0.0002  (-0.0003, -0.00006) | -0.00009  (-0.0002, 0.00002) | -0.0001  (-0.0002, -0.00007) | 0.0003  (-0.0001, 0.0008) | -0.0003  (-0.0009, 0.0001) |
| Total PCB (ppb) | -0.0007  (-0.0509, 0.0494) | 0.0200  (-0.0292, 0.0692) | 0.0142  (-0.0307, 0.0591) | -0.0150  (-0.0466, 0.0166) | 0.0492  (-0.1267, 0.2251) | -0.1093  (-0.3109, 0.0921) |

**Table S6: Association between PBB and thyroid hormone levels in each gender**

|  | Women (N = 440) | | Men (N = 275) | | Interaction | |
| --- | --- | --- | --- | --- | --- | --- |
|  | T-statistic | P-value | T-statistic | P-value | T-statistic | P-value |
| Total T_4_ ($\mu$g/dL) | 0.87 | 0.38 | 0.58 | 0.55 | 0.29 | 0.76 |
| Total T_3_ (ng/mL) | 1.25 | 0.21 | 0.89 | 0.37 | -0.07 | 0.94 |
| Free T_4_ (ng/dL) | -2.27 | 0.02 | -1.45 | 0.14 | 0.14 | 0.88 |
| Free T_3_ (pg/mL) | 2.98 | 0.002 | 0.94 | 0.34 | -1.73 | 0.08 |
| TSH ($\mu$IU/mL) | 0.90 | 0.36 | 1.04 | 0.29 | -0.17 | 0.85 |
| Free T_3_: Free T_4_ ratio | 3.69 | 0.0002 | 1.68 | 0.09 | -0.98 | 0.32 |

**Table S7: Association between PCB and thyroid hormone levels in each gender**

|  | Women (N = 440) | | Men (N = 275) | | Interaction | |
| --- | --- | --- | --- | --- | --- | --- |
|  | T-statistic | P-value | T-statistic | P-value | T-statistic | P-value |
| Total T_4_ ($\mu$g/dL) | 1.86 | 0.06 | -1.20 | 0.23 | -1.18 | 0.23 |
| Total T_3_ (ng/mL) | 1.76 | 0.07 | 0.55 | 0.57 | -0.57 | 0.56 |
| Free T_4_ (ng/dL) | 4.17 | 3.57e-05 | 0.31 | 0.75 | -2.15 | 0.03 |
| Free T_3_ (pg/mL) | -1.41 | 0.15 | 0.97 | 0.33 | 0.40 | 0.68 |
| TSH ($\mu$IU/mL) | -0.82 | 0.40 | -1.25 | 0.21 | -0.59 | 0.55 |
| Free T_3_: Free T_4_ ratio | -4.29 | 2.19e-05 | 0.40 | 0.68 | 2.03 | 0.04 |

**Table S8: Regression coefficients from the association of PBB exposure and thyroid hormone levels subset by quartile of age of exposure to PBB**

|  | First quartile (Age of exposure < 2.6 years); N = 179 | | | | | |
| --- | --- | --- | --- | --- | --- | --- |
|  | Total T_4_ ($\mu$g/dL) | Total T_3_ (ng/mL) | Free T_4_ (ng/dL) | Free T_3_ (pg/mL) | TSH ($\mu$IU/mL) | Free T_3_: Free T_4_ ratio |
| Variables | $\beta$  (95% CI) | $\beta$  (95% CI) | $\beta$  (95% CI) | $\beta$  (95% CI) | $\beta$  (95% CI) | $\beta$  (95% CI) |
| Total PBB (ppb) | 0.0101  (-0.0083, 0.0285) | 0.0138  (-0.0115, 0.0391) | -0.0100  (-0.0294, 0.0094) | 0.0189  (0.0015, 0.0364) | 0.0306  (-0.0377,0.0991) | 0.1283  (0.0335, 0.2232) |
| Current Age (years) | -0.0013  (-0.0060, 0.0033) | -0.0103  (-0.0167, -0.0039) | -0.0024  (-0.0073, 0.0024) | -0.0041  (-0.0085, 0.0002) | -0.0075  (-0.0248,0.0097) | -0.0124  (-0.0363, 0.0115) |
| Sex | -0.0146  (-0.2325, -0.0597) | -0.0535  (-0.1721, 0.0651) | 0.0151  (-0.0758, 0.1061) | 0.0691  (-0.0126, 0.1510) | 0.2123  (-0.1082, 0.5329) | 0.1668  (-0.2775, 0.6112) |
| Lipids (mg/dL) | -0.0001  (-0.0002, -0.000008) | -0.00008  (-0.0002, 0.00009) | -0.0001  (-0.0003, -0.00005) | -0.0001  (-0.0002, 0.000009) | 0.0001  (-0.0003, 0.0006) | 0.0004  (-0.0002, 0.0010) |
| Total PCB (ppb) | 0.0102  (-0.0175, 0.0380) | 0.0210  (-0.0170, 0.0592) | 0.0503  (0.0210, 0.0795) | -0.0038  (-0.0301, 0.0224) | -0.0832  (-0.1863, 0.0197) | -0.1978  (-0.3406, -0.0550) |
|  | Second quartile (Age of exposure > 2.6 years & < 10.94 years); N = 178 | | | | | |
|  | Total T_4_ ($\mu$g/dL) | Total T_3_ (ng/mL) | Free T_4_ (ng/dL) | Free T_3_ (pg/mL) | TSH ($\mu$IU/mL) | Free T_3_: Free T_4_ ratio |
| Variables | $\beta$  (95% CI) | $\beta$  (95% CI) | $\beta$  (95% CI) | $\beta$  (95% CI) | $\beta$  (95% CI) | $\beta$  (95% CI) |
| Total PBB (ppb) | 0.0064  (-0.0137, 0.0267) | 0.0063  (-0.0146, 0.0272) | -0.0036  (-0.0203, 0.0131) | 0.0048  (-0.0085, 0.0181) | -0.0330  (-0.1107,0.0446) | 0.0298  (-0.0586, 0.1183) |
| Current Age (years) | -0.0065  (-0.0134, 0.0002) | -0.0162  (-0.0232, -0.0091) | 0.0007  (-0.0049, 0.0063) | -0.0091  (-0.0136, -0.0047) | -0.0202  (-0.0464,0.0058) | -0.0400  (-0.0698, -0.0102) |
| Sex | -0.0040  (-0.0666, 0.0586) | 0.0376  (-0.0271, 0.0102) | 0.0076  (-0.0441, 0.0593) | 0.0855  (0.0442, 0.1267) | 0.2065  (-0.0336, 0.4467) | 0.3402  (0.0667, 0.6137) |
| Lipids (mg/dL) | 0.00007  (-0.00005, 0.0002) | -0.0001  (-0.0003, -0.00004) | -0.00002  (-0.0001, 0.00008) | -0.0001  (-0.0001, -0.00001) | 0.0001  (-0.0003, 0.0007) | -0.0003  (-0.0009, 0.0002) |
| Total PCB (ppb) | 0.0198  (-0.0178, 0.0575) | 0.0432  (0.0041, 0.0822) | 0.0127  (-0.0184, 0.0439) | 0.0010  (-0.0139, 0.0356) | 0.0432  (-0.1014, 0.1879) | -0.0186  (-0.1834, 0.1460) |
|  | Third quartile (Age of exposure > 10.94 years & <22.38 years); N = 179 | | | | | |
|  | Total T_4_ ($\mu$g/dL) | Total T_3_ (ng/mL) | Free T_4_ (ng/dL) | Free T_3_ (pg/mL) | TSH ($\mu$IU/mL) | Free T_3_: Free T_4_ ratio |
| Variables | $\beta$  (95% CI) | $\beta$  (95% CI) | $\beta$  (95% CI) | $\beta$  (95% CI) | $\beta$  (95% CI) | $\beta$  (95% CI) |
| Total PBB (ppb) | -0.0181  (-0.0390, 0.0027) | -0.0088  (-0.0332, 0.0155) | -0.0234  (-0.0446, -0.0022) | -0.0032  (-0.0174, 0.0109) | 0.0615  (-0.0148,0.1380) | 0.0758  (-0.0308, 0.1824) |
| Current Age (years) | -0.00004  (-0.0079, 0.0078) | -0.0033  (-0.0126, 0.0059) | 0.0004  (-0.0076, 0.0084) | -0.0001  (-0.0055, 0.0052) | 0.0068  (-0.0221,0.0358) | -0.0063  (-0.0468, 0.0340) |
| Sex | 0.0030  (-0.0562, 0.0624) | 0.0096  (-0.0596, 0.0789) | 0.0458  (-0.0142, 0.1058) | 0.0444  (0.0041, 0.0847) | -0.1716  (-0.3884, 0.0452) | -0.0025  (-0.3051, 0.3000) |
| Lipids (mg/dL) | -0.00007  (-0.0002, 0.00007) | -0.0001  (-0.0003, -0.00002) | 0.00002  (-0.0001, 0.0001) | -0.00007  (-0.0001, 0.00002) | 0.00008  (-0.0004, 0.0006) | -0.0004  (-0.0011, 0.0003) |
| Total PCB (ppb) | 0.0016  (-0.0354, 0.0322) | 0.0065  (-0.0329, 0.0460) | 0.0193  (0.0148, 0.0536) | -0.0066  (-0.0296, 0.0162) | -0.0208  (-0.1445, 0.1028) | -0.1082  (-0.2807, 0.0643) |
|  | Fourth quartile (Age of exposure > 22.37 years); N = 179 | | | | | |
|  | Total T_4_ ($\mu$g/dL) | Total T_3_ (ng/mL) | Free T_4_ (ng/dL) | Free T_3_ (pg/mL) | TSH ($\mu$IU/mL) | Free T_3_: Free T_4_ ratio |
| Variables | $\beta$  (95% CI) | $\beta$  (95% CI) | $\beta$  (95% CI) | $\beta$  (95% CI) | $\beta$  (95% CI) | $\beta$  (95% CI) |
| Total PBB (ppb) | 0.0053  (-0.0155, 0.0262) | -0.0047  (-0.0265, 0.0171) | -0.0053  (-0.0246, 0.0013) | 0.0001  (-0.0138, 0.0142) | 0.0103  (-0.0618,0.0825) | 0.0205  (-0.0648, 0.1059) |
| Current Age (years) | -0.0008  (-0.0058, 0.0041) | -0.0095  (-0.0147, -0.0043) | 0.0041  (-0.0004, 0.0087) | -0.0063  (-0.0096, -0.0030) | 0.0151  (-0.0019,0.0323) | -0.0372  (-0.0575, -0.0169) |
| Sex | -0.0533  (-0.1193, 0.0126) | -0.0360  (-0.1050, 0.0329) | -0.0357  (-0.0968, 0.0253) | 0.0089  (-0.0354, 0.0533) | 0.0777  (-0.1506, 0.3061) | 0.1512  (-0.1188, 0.4214) |
| Lipids (mg/dL) | -0.0001  (-0.0002, 0.00005) | -0.0002  (-0.0003, -0.00005) | -0.0001  (-0.0002, 0.00003) | -0.0002  (-0.0003, -0.0001) | 0.0005  (0.000006, 0.0010) | -0.0005  (-0.0012, 0.00006) |
| Total PCB (ppb) | 0.0051  (-0.0377, 0.0481) | 0.0076  (-0.0372, 0.0525) | 0.0199  (-0.0198, 0.0596) | -0.0035  (-0.0324, 0.0253) | -0.0734  (-0.2220, 0.0751) | -0.0789  (-0.2547, -0.0967) |

**Table S9: Regression coefficients from the association of PBB exposure and thyroid hormone levels subset by median of age of exposure to PBB**

|  | Below median (Age of exposure < 10.94 years) | | | | | |
| --- | --- | --- | --- | --- | --- | --- |
|  | Total T_4_ ($\mu$g/dL) | Total T_3_ (ng/mL) | Free T_4_ (ng/dL) | Free T_3_ (pg/mL) | TSH ($\mu$IU/mL) | Free T_3_: Free T_4_ ratio |
| Variables | $\beta$  (95% CI) | $\beta$  (95% CI) | $\beta$  (95% CI) | $\beta$  (95% CI) | $\beta$  (95% CI) | $\beta$  (95% CI) |
| Total PBB (ppb) | 0.0136  (0.0014, 0.0258) | 0.0211  (0.0062, 0.0359) | -0.0079  (-0.0193, -0.0035) | 0.0187  (0.0088, 0.0286) | 0.0235  (-0.0219,0.0690) | 0.1082  (0.0501, 0.1661) |
| Current Age (years) | -0.0020  (-0.0050, 0.0009) | -0.0089  (-0.0126, -0.0053) | -0.0014  (-0.0042, 0.0013) | -0.0035  (-0.0059, -0.0011) | -0.0012  (-0.0124,0.0098) | -0.0127  (-0.0269, 0.0013) |
| Sex | -0.0590  (-0.1093, -0.0087) | -0.0054  (-0.0665, 0.0557) | -0.0126  (-0.0346, 0.0599) | 0.0696  (0.0288, 0.1105) | 0.1721  (-0.0156, 0.3598) | 0.2323  (-0.0068, 0.4715) |
| Lipids (mg/dL) | -0.00004  (-0.0001, 0.00004) | -0.0001  (-0.0002, -0.00004) | -0.0001  (-0.0001, -0.00002) | -0.0001  (-0.0002, -0.00005) | 0.0001  (-0.0002, 0.0004) | -0.00002  (-0.0004, 0.0004) |
| Total PCB (ppb) | 0.0113  (-0.0108, 0.0334) | 0.0268  (-0.00007, 0.0538) | 0.0382  (0.0174, 0.0591) | -0.0009  (-0.0189, 0.0171) | -0.0444  (-0.1272, 0.0383) | -0.1474  (-0.2528, -0.0419) |
|  | Above median (Age of exposure > 10.94 years) | | | | | |
|  | Total T_4_ ($\mu$g/dL) | Total T_3_ (ng/mL) | Free T_4_ (ng/dL) | Free T_3_ (pg/mL) | TSH ($\mu$IU/mL) | Free T_3_: Free T_4_ ratio |
| Variables | $\beta$  (95% CI) | $\beta$  (95% CI) | $\beta$  (95% CI) | $\beta$  (95% CI) | $\beta$  (95% CI) | $\beta$  (95% CI) |
| Total PBB (ppb) | -0.0048  (-0.0194, 0.0097) | -0.0059  (-0.0220, 0.0102) | -0.0127  (-0.0268, 0.0014) | -0.0001  (-0.0101, 0.0098) | 0.0301  (-0.0217,0.0821) | 0.0465  (-0.0202, 0.1133) |
| Current Age (years) | 0.0007  (-0.0016, 0.0031) | -0.0053  (-0.0079, -0.0026) | 0.0029  (0.0006, 0.0052) | -0.0030  (-0.0046, -0.0013) | 0.0046  (-0.0039,0.0131) | -0.0224  (-0.0334, -0.0114) |
| Sex | -0.0281  (-0.0718, 0.0155) | -0.0119  (-0.0602, 0.0362) | 0.0020  (-0.0402, 0.0443) | 0.0265  (-0.0033, 0.0564) | -0.0387  (-0.1943, 0.1168) | 0.0830  (-0.1169, 0.2830) |
| Lipids (mg/dL) | -0.00009  (-0.0002, 0.000008) | -0.0002  (-0.0003, -0.00009) | -0.00005  (-0.0001, 0.00005) | -0.0001  (-0.0002, -0.0001) | 0.0003  (-0.00001, 0.0007) | -0.0004  (-0.0009, -0.00001) |
| Total PCB (ppb) | 0.0008  (-0.0255, 0.0272) | 0.0062  (-0.0228, 0.0354) | 0.0212  (-0.0043, 0.0467) | -0.0064  (-0.0245, 0.0116) | -0.0443  (-0.1382, 0.0496) | -0.1033  (-0.2241, 0.0174) |
